# Enhancing Dementia Care in Primary Care: Impact of Targeted Training and Electronic Medical Record (EMR)-Integrated Algorithms

**DOI:** 10.7759/cureus.107031

**Published:** 2026-04-14

**Authors:** Daniel D Sewell, Gene Kallenberg, Barbara Mandel, Ammar Mandvi, Lisa Asmus, Ian C Neel, Graham Heimler, William Andrew, Michael Lobatz

**Affiliations:** 1 Psychiatry, University of California San Diego, San Diego, USA; 2 Family Medicine, University of California San Diego, San Diego, USA; 3 Champions for Health, San Diego County Medical Society Foundation, San Diego, USA; 4 School of Public Health, San Diego State University, San Diego, USA; 5 Medicine, University of California San Diego, San Diego, USA; 6 Neurology, Scripps Health, San Diego, USA; 7 Neurology, The Neurology Center of Southern California, San Diego, USA

**Keywords:** alzheimer’s disease, cognitive impairment, cognitive screening, dementia, early detection, family medicine, older adults, primary care

## Abstract

Introduction: The number of medical specialists whose training programs provide robust education in dementia diagnosis and treatment pales in comparison to the increasing number of individuals living with dementia. Primary care providers (PCPs) care for most older adults with cognitive concerns and dementia. Summarized here are the results of an effort to help PCPs care for these patients using targeted training and electronic medical record (EMR)-integrated clinical algorithms.

Methods: Clinicians from two University of California San Diego Family Medicine Clinics completed assessments of dementia knowledge and comfort in caring for patients and family members impacted by dementia four times: enrollment, and approximately two, nine and 15 months after a three-component intervention: 1) training via four online educational modules (six hours total) on screening, evaluation, and care of patients with dementia; 2) integration of clinical algorithms into the EMR system and 3) access to mentoring from a more experienced peer.

Results: Subjective assessment of comfort and competence of intervention group clinicians in working with patients with cognitive complaints significantly increased and was higher for intervention group PCPs than the comparison group PCPs. Evidence of completed AD8s (Eight-Item Informant Interview to Differentiate Aging and Dementia) at both clinics increased from baseline to post-intervention: 52.85% to 82.6% and 66.1% to 86.9%.

Discussion: Training PCPs on dementia screening and diagnosis, and integration of algorithms into the EMR, improved clinician subjective competence and comfort in caring for patients with cognitive complaints and increased the AD8 completion rate. The small number of study participants mandates caution when interpreting these findings.

## Introduction

A substantial portion of those who would meet the diagnostic criteria for Alzheimer’s or a related dementia have not been diagnosed with dementia by a physician [1]. Although licensed primary care providers (LPCPs) are often the first among healthcare providers to encounter patients with cognitive concerns or to observe functional losses resulting from cognitive impairment [2], they may be the most challenged to provide optimal care while also meeting other clinical demands and clinical productivity expectations.

The mandated components of the Medicare Annual Wellness Visit (AWV) include an assessment of an individual’s cognition, but do not specify screening instruments or a workflow [3]. AWV guidelines do emphasize, however, the importance of direct observation by the clinician and inclusion of historical information from corroborative sources. The AWV is underused and experienced by only 23 to 34% of those eligible [4-5]. When AWVs do occur, fewer than 30% include a cognitive assessment [6]. In addition, only about half of Medicare beneficiaries who have a diagnosis of Alzheimer’s or another dementia in their Medicare billing records report being informed of the diagnosis [7-11]. Improving this situation will require solutions that help LPCPs identify and care for those with cognitive concerns with both precision and efficiency. The provision of targeted training and the integration of clinical algorithms into the electronic medical record (EMR) are two recommended solutions.

The San Diego County Alzheimer’s Project (SDCAP) is an ongoing regional initiative established by the San Diego County Board of Supervisors in 2014 to address the toll of Alzheimer’s disease (AD) and related dementias on county residents and healthcare systems [12]. Because LPCPs are often the first healthcare professionals to encounter cognitive impairment among older adults [2], the Clinical Roundtable (CR) of the SDCAP sought to enhance dementia care in the primary care setting through the development and dissemination of best practice guidelines for PCPs, accompanied by a variety of educational resources, including live and on-demand lectures. The CR, composed of dementia care experts from all of the major health systems in San Diego County (SDC), periodically updates the guidelines and resources. The fourth edition of the guidelines was published in June 2024 [13].

A study was launched in 2022 to assess the impact of guideline-based educational efforts and the EMR integration of clinical algorithms on clinician comfort and competence caring for patients with cognitive complaints and on the rate of dementia screening. This article summarizes the study and its results.

## Materials and methods

Study hypothesis

**Figure 1:**
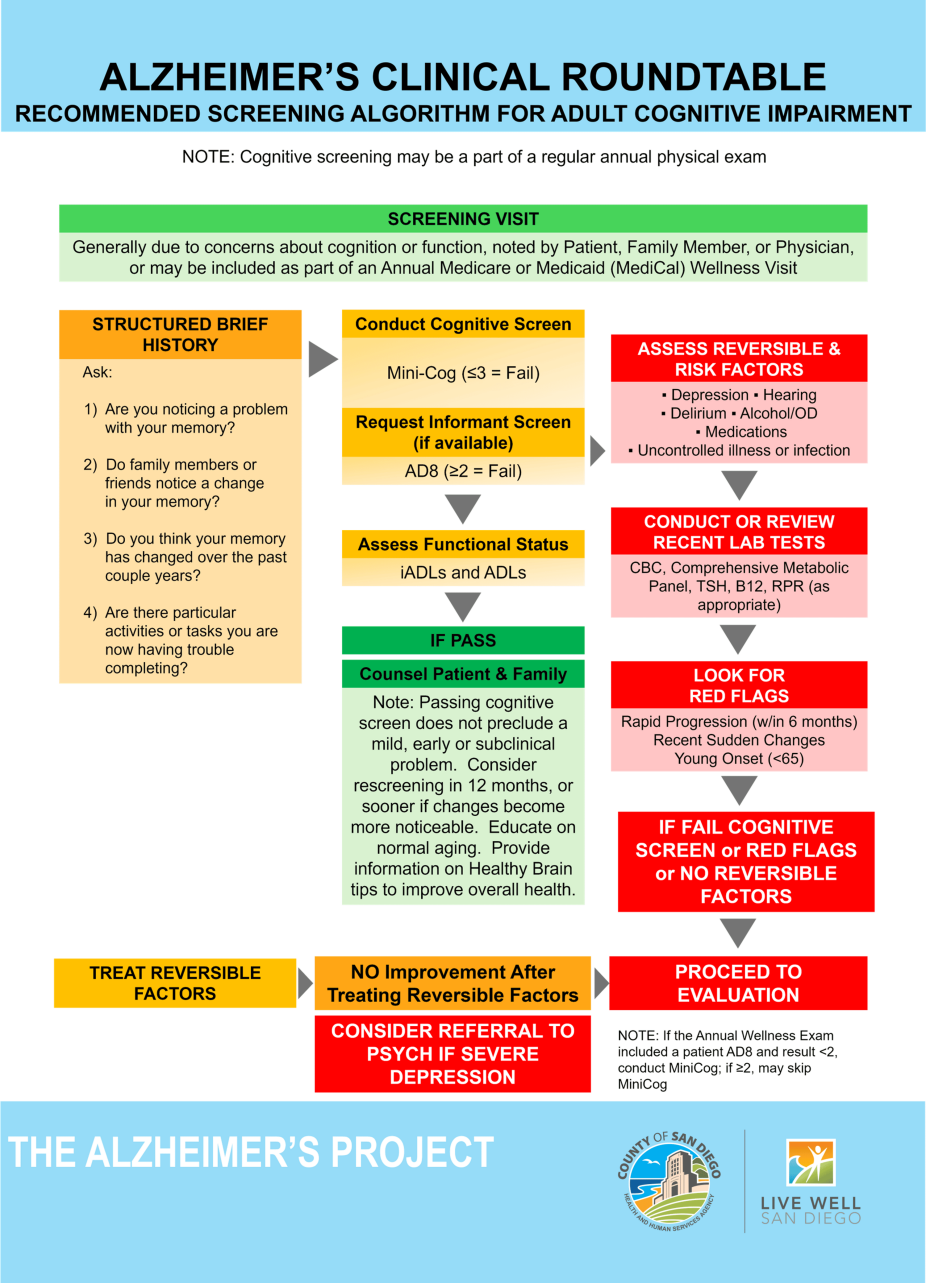
SDCAP's screening algorithm The Alzheimer's Project's recommended screening algorithm for adult cognitive impairment was created by the San Diego Alzheimer's Project Clinical Roundtable for the fourth edition of the Clinical Roundtable Guidelines for Screening, Evaluation, and Diagnosis of Alzheimer's Disease [13]. Microsoft PowerPoint (Microsoft Corp., Redmond, WA, USA) was used for the creation of the algorithm design. SDCAP: San Diego County Alzheimer’s Project, iADL: instrumental activities of daily living, ADL: activities of daily living, TSH: thyroid-stimulating hormone, RPR: rapid plasma reagin

After a 12-month period of targeted training and integration of the SDCAP’s clinical algorithms into the EMR, PCPs will self-report increased comfort and competence in screening and caring for patients with cognitive concerns and their family caregivers. The SDCAP’s dementia screening algorithm appears in Figure 1.

Baseline clinical information

Baseline Clinical Data Collection

Immediately after receiving IRB approval, the study coordinator collected baseline data from the clinics participating in the study. This baseline data included the number of patients over 65 years in the two participating clinics and the racial composition of each clinic. The racial composition of the two clinics differed (Table 1).

**Table 1:** Description of patient populations of the family medicine clinics N=3,416 (100%) ^a ^Difference in proportion of White, Asian, Black, or Other races calculated using the Chi-squared test with 3 degrees of freedom. ^b^ Includes Hispanic.

Clinic	First intervention clinic (Clinic 1) n (%)	Second intervention clinic (Clinic 2) n (%)	Statistical test^a^	
			X^2^	p
Number of patients 65-years-old or older in 2021	1,616 (100)	1,800 (100)	-	-
Race	-	-	97.61	< .001
-White/Caucasian^b^	889 (55)	1224 (68)	-	-
-Asian	436 (27)	252 (14)	-	-
-Other	226 (14)	270 (15)	-	-
-Black	65 (4)	54 (3)	-	-
Ethnicity	-	-	-	-
Hispanic	129 (8)	180 (10)	-	-

Preparation of the Clinics and Recruitment of Licensed Professionals

The study coordinator met with the LPCPs and administrative staff at each clinic to explain the study, including the role of the participating LPCPs, and obtained informed consent from the participating LPCPs. Clinicians participating throughout the course of the study and who completed all necessary evaluation surveys received a financial incentive after submission of the last research survey and completion of the qualitative interview.

Study population

Licensed primary care providers (LPCPs) working in two UC San Diego Health (UCSDH) Family Medicine Clinics.

Inclusion Criteria

LPCPs working at either of the two UCSDH Family Medicine Outpatient Clinics. LPCPs may include Medical Doctors (MDs), Doctors of Osteopathic Medicine (DOs), residents, Physician Assistants (PAs), and Registered Nurse Practitioners (RNPs).

Exclusion Criteria

Failure to complete the required training.

**Figure 2:**
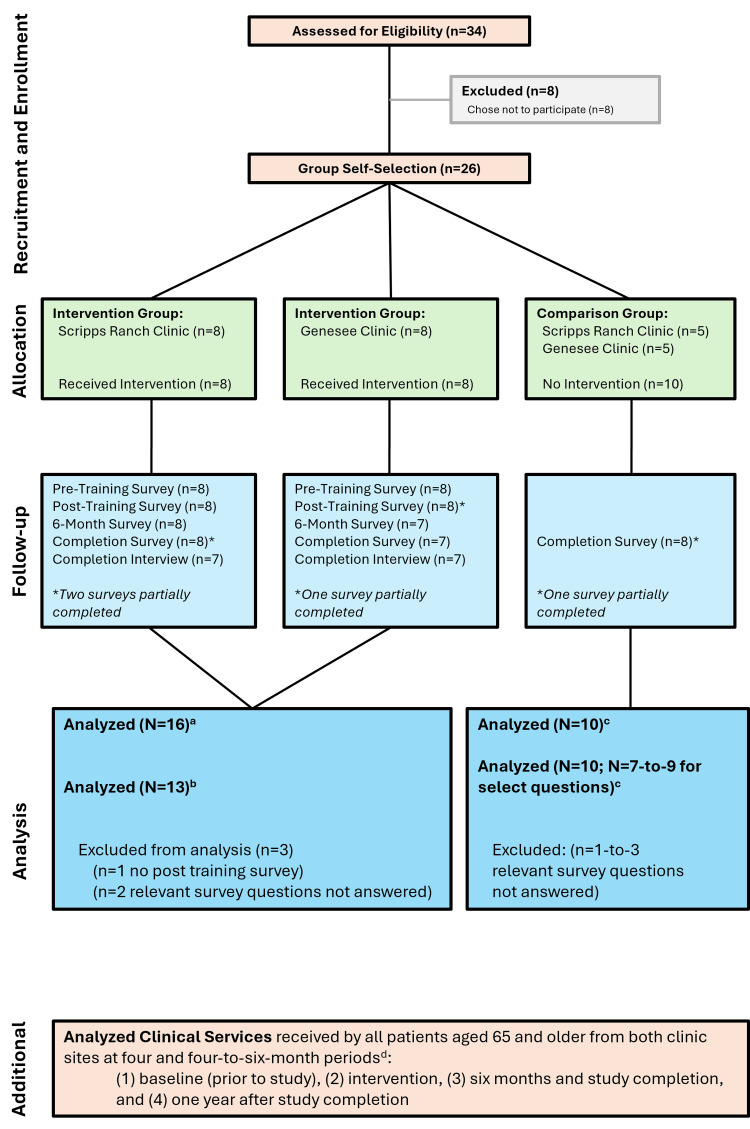
Cohort flowchart LPCP: licensed primary care providers, SDCAP: San Diego County Alzheimer’s Project ^a^ See "Results" section subheading "Clinician initial questionnaire (LPCP intervention group)" and "Self-reported clinical practices: enrollment and post-training (LPCP intervention group)." ^b^ See "Results" section subheading "Self-reported clinical practices: enrollment and post-training (LPCP intervention group)," "Self-reported clinical practices over time: enrollment, post-training, interval, and completion (LPCP intervention group)," and "Self-reported clinical practice: LPCP intervention and LPCP comparison group." ^c^ See "Results" section subheading "Self-reported clinical practice: LPCP intervention and LPCP comparison group." ^d^ See "Results" section subheading "Assessment of clinician adherence to the SDCAP screening algorithm." Figure created using Microsoft PowerPoint (Microsoft Corp., Redmond, WA, USA).

The 16 (100%) LPCPs who agreed to complete the required training in dementia screening and diagnosis formed the intervention group and included MDs (12, 75.0%), resident physicians (3, 18.8%), PAs and RNPs (1, 6.3%). An additional 10 LPCPs who did not receive the intervention served as a comparison group (N=10, 100%). Figure 2 depicts the intervention and comparison groups along with follow-up and analysis information.

Intervention

To enhance learning and use of the guidelines and other resources, a clinician in each clinic was designated as the “clinic champion.” This clinician had access to ongoing mentoring sessions and consultations from the clinical educators, a pool of dementia specialists, all of whom were members of the SDCAP CR. All participating LPCPs were required to engage in four one-to-1½- hour continuing medica education (CME) accredited on-demand webinars with the following themes: screening for dementia; evaluation and diagnosis of dementia; treating the common behavioral problems experienced by individuals living with dementia; and life planning through the stages of dementia including available community resources to support individuals living with dementia and their family members. Clinicians also received printed and online resource materials and access to a clinic champion to answer questions and support learning [14].

Preparation of the Electronic Medical Record (EMR)

UCSDH uses the electronic medical record system EPIC (Epic Systems Corporation, Verona, WI, USA). Some changes that supported this study had been made to EPIC prior to the initiation of the study. For example, several years prior to the launch of this study, enhancements made to EPIC included the addition of the participant-completed AD8 [15] to the Medicare AWV, which had been added to the Health Risk Appraisal. To better support this study, EPIC was modified so that the AD8 was added to any clinic visit, not just the AWV. There were two reasons for this: 1) a substantial number of patients do not make or keep their AWV; and 2) the goal of screening the clinic population of the two study clinics in a shorter period of time than would have occurred if screening of all of the 65 years-old and older patients had only been done during the AWV since many of these patients visit the clinic several times a year.

**Figure 3:**
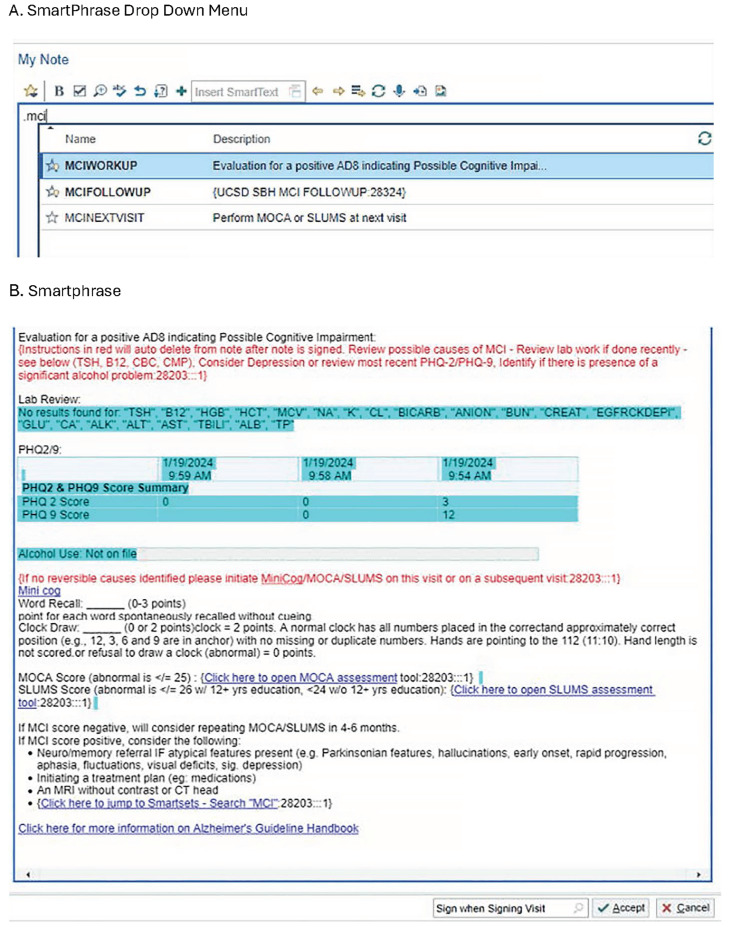
Examples of software additions to EPIC created for this study A) Upon entering .mci EPIC (Epic Systems Corporation, Verona, WI, USA), users are provided Smartphrase options for suggested workup of abnormal AD8 screens, follow-up visit recommendations, and information on steps recommended to be performed at the patient's next visit. B) The Smartphrase for .mciworkup is displayed, revealing the recommended evaluation with links to tools within EPIC for cognitive assessment tools, algorithm for response to cognitive assessment scores, and a link to the Alzheimer's Project Clinical Roundtable Guidelines [13] for more detailed information on cognitive evaluation recommendations.

Prior to the initiation of this research, additional efforts were taken to leverage the EPIC software to facilitate clinician adherence to SDCAP’s dementia screening and diagnosis algorithms. Numerous additional modifications were made to EPIC to facilitate clinician adherence to the SDCAP’s dementia screening and diagnosis algorithms. Smart phrases (containing automated documentation and just-in-time decision support), Smartsets (automated order lists), banners with suggested actions, embedded links to helpful resource information for clinicians (e.g., the digital version of the guidelines), and embedded links to add resource information for caregivers into the After Visit Summary (AVS) were created (Figure 3). For patients with a self-reported AD8 score suggesting the possibility of a cognitive problem (>=2), an automatically triggered banner was created to prompt further assessment. Based on feedback from participating LPCPs at Clinic 1 (Scripps Ranch), additional EPIC refinements were made after initiation of the study, which improved banner performance. The banner stated "Patient has a positive screen for possible cognitive impairment. Please perform a Mini-Cog/MoCA/SLUMs today or at a future visit (if future invite family member to come). Use .mciworkup in your progress note to provide additional guidance. You can use .mcinextvisit if you are inviting the patient to return for evaluation." 

For patients with an AD8 score suggesting the presence of a cognitive problem (AD8>=2), banners indicating the need for further assessment were created to appear automatically. The banners suggest a Montreal Cognitive Assessment (MoCA) [16] or the Saint Louis University Mental Status Examination (SLUMS) [17] during the same visit or at a follow-up visit scheduled specifically for this purpose (Figure 3). The banners also listed Smartphrases, which were created with links to past relevant labs, MoCA/SLUMS flowsheet recording fields, and decision support for patients having certain characteristics identified in the SDCAP Guidelines as “Red Flags” (e.g., rapid symptom progression or recently emerged changes in motor function). The Red Flags suggest prompt referral to a neurologist or neuropsychologist for further evaluation, including cognitive testing. The banners also had links to Smartsets for follow-up actions by PCPs. Figure 3 provides examples of EPIC software additions created for this study. Lastly, a few changes based on learned experience were made prior to participating clinicians in the second intervention clinic experiencing the study interventions. (e.g., assuring the banner continues firing until the MoCA/SLUMS is completed, and reprogramming so that AD8 and banner pop-ups are not triggered for patients once given a dementia diagnosis).

Data collection instruments for subjective outcomes

LPCPs completed all research tasks/assessment instruments online. All instruments are included in the supplemental information (Appendix A and Appendix B).

Clinician Initial Questionnaire (CIQ)

This questionnaire was developed by the authors and is available in the supplemental information (Appendix A). The questionnaire was completed at the time of enrollment and, in addition to demographic information and questions about participating in previous dementia trainings, including an assessment of subjective outcomes regarding learning experiences provided through the SDCAP, contained five questions that were similar to questions included in the Clinical Practice Survey (described below), which were used to establish clinician comfort and competence at baseline.

Clinical Practice Survey (CPS)

This survey was developed by the authors and is available in supplemental information (Appendix B). LPCPs in the intervention group completed this instrument at three time points: Post-training (approximately two months after enrollment), Interval (nine to 10 months after enrollment), and completion (13 to 16 months after enrollment). LPCPs in the comparison group completed the CPS only once, 13 to 16 months after their intervention group peers enrolled in the study.

Each of the five similar questions in the CIQ and CPS required the selection of one out of five possible answers on a rating scale. All questionnaires/surveys were completed online. Clinicians who completed all required research tasks received a financial incentive.

Statistical analysis

The surveys were conducted using a confidential REDCap instrument, a survey data collection tool developed by Vanderbilt University and utilized by more than 7,200 institutions (https://projectredcap.org/resources/videos/). Participants were coded with a unique identifier so that the statistician/evaluator did not know the identity of the individual. Data was downloaded for analysis in Excel (Microsoft Corp., Redmond, WA, USA)/CSV and then converted to IBM SPSS Statistics for Windows, version 30 (IBM Corp., Armonk, NY, USA). The study was organized as a time series design in which both clinics received the intervention within the study period, in a timed implementation model.

A comparison of LPCP CIQ/CPS responses over time utilized a non-parametric Wilcoxon Signed-Rank test to account for the small sample size and matching of data by participant. The Mann-Whitney U test was utilized to test for differences between the intervention and comparison LPCP groups. The Pearson Chi-square test was utilized to test for differences in dementia screening rates over time.

## Results

Clinician initial questionnaire (LPCP intervention group)

Sixteen LPCPs enrolled in the intervention and completed a CIQ prior to the initiation of the intervention (Table 2). Eight practiced at Clinic 1 and eight at Clinic 2. In addition to demographics and previous training, LPCPs were asked about their use of MoCA/SLUMS assessment as well as their use of the Mini-Mental State Examination (MMSE) [18], Mini-Cog [19], and the Eight-Item Informant Interview to Differentiate Aging and Dementia (AD8).

**Table 2:** Clinical initial questionnaire responses at baseline N=16 (100%). UCSD: University of California San Diego, SDCAP: San Diego County Alzheimer’s Project ^a^ Three persons who responded that they had not used the SDCAP Clinical Guidelines indicated specific guidelines they had used: use of MoCA, SLUMS, or MMSE (n=2, 66%), screening algorithm (n=1, 33%), Mini-Cog and/or AD8 (n=1, 33%), depression screening (n=1, 33%), and behavioral management (n=1, 33%). ^b^ Montreal Cognitive Assessment (MoCA); Saint Louis University Mental Status (SLUMS); Mini-Mental State Examination (MMSE); Mini-Cog; Eight-Item Informant Interview to Differentiate Aging and Dementia (AD8).

Question number	Question	Response	n	%
1.	Received training on dementia in the past four years	Yes (includes one who responded ‘no’ but answered subsequent questions)	13	81.3
		No	3	18.8
1a.	If yes, most recent training (n=13, 100% responding)	Within the past month	1	7.7
		2-6 months ago	4	30.8
		7-12 months ago	2	15.4
		More than 1 year ago	5	38.5
		Not specified	1	7.7
1b.	If yes, training subject(s) (n=13, 100% responding)	Pharmacotherapy	8	61.5
		Screening patients with cognitive declines	8	61.5
		Evaluation and diagnosis	7	53.8
		Program overview	2	15.4
		Behavioral management	2	15.4
		Pharmacological management of behavioral issues	2	15.4
		End-of-life issues	2	15.4
		Patient and caregiver communications	0	0.0
		Mindfulness/self-care	0	0.0
1c.	If yes, training method(s) (n=13, 100% responding)	Grand rounds/presentations live webinar or in-person at my health system	6	46.2
		Professional conference/meeting	4	30.8
		Online recorded webinar	2	15.4
		Other	2	15.4
		Reviewed championsforhealth.org/alzheimers website	1	7.7
2.	Participated in the UCSD Center for Mindfulness program	Yes	1	6.3
3.	Perform the Medicare Annual Wellness Visit	Yes	16	100.0
4.	Conduct screening on patients for suspected dementia	Yes	16	100.0
5.	How often screen for dementia among patients 65 and older	As part of an annual wellness exam	13	81.3
		When the patient or family member requests	3	18.8
6.	How often do your patients that you screen require a complete evaluation for dementia?	About 10% of patients screened	9	56.3
		About 20% of patients screened	1	6.3
		About 50% of patients screened	0	0.0
		About 75% of patients screened	1	6.3
		Unsure	5	31.3
7.	Conduct evaluation or refer to specialist?	Refer to specialist	11	68.8
		Conduct evaluation myself	5	31.3
8.	What percent of patients visits are with patients 65+ in an average month	11-25%	6	37.5
		26-50%	9	56.3
		50%-75%	1	6.3
9.	Incorporated any of the SDCAP Clinical Guidelines in your practice?	Yes and/or indicated frequency used	8	50.0
9a.	If yes, which ones? (n=11, 100% responding)^a^	Use of MoCA,^b^ SLUMS,^b^ or MMSE instrument^b^	9	56.3
		Depression screening	8	50.0
		Use of Mini-Cog^b^ and/or AD8^b^	6	37.5
		Pharmacological management of behavioral signs and symptoms	3	18.8
		Screening algorithm	2	12.5
		Life planning through the stages of disease, including end-of-life planning	2	12.5
		Behavioral management	2	12.5
		Evaluation algorithm	0	0.0
		Informant surveys	0	0.0
10.	Number of times used SDCAP Clinic Guidelines (other than screening for depression) (n=13, 100% responding)	Never	7	53.8
		1-5 times	3	23.1
		6-15 times	1	7.7
		More than 15 times	2	15.4
11.	Professional designation	MD	12	75.0
		Medical Resident	3	18.8
		NP (Nurse Practitioner)	1	6.3
12.	Preferred gender	Female	10	62.5
		Male	6	37.5
13.	Length in practice (Primary Care Provider)	Less than 5 years	6	37.5
		Five to 10 years	2	12.5
		More than 10 years	8	50.0
14.	Ethnicity	White	11	68.8
		Asian	3	18.8
		Hispanic or Latino/a	1	6.3
		Black or African-American and Hispanic/Latino(a)	1	6.3
15.	Approximate age in 2023	25-30 years	3	18.8
		31-40 years	7	43.8
		51-60 years	6	37.5
16.	‘Extremely’ or ‘Moderately’ comfortable with the following after training in the past (or currently if not previously trained). (number and percent extremely or moderately comfortable)	Communicating with patients and family caregivers on dementia	6	37.5
		Screening for dementia	5	31.3
		Diagnosing dementia	4	25.0
		Making referrals to community resources	3	18.8
		Treating behavioral symptoms	3	18.8

Self-reported clinical practices: enrollment and post-training (LPCP intervention group)

The initial survey completed by LPCPs at enrollment asked, in addition to other questions, about the practitioner’s comfort levels. This provided the enrollment, or baseline, data. LPCPs in the intervention group completed the CPS after the educational intervention concluded, which was approximately two months after enrollment (post-training). Five of the questions on the CPS were similar to those asked at enrollment on the CIQ.

**Figure 4:**
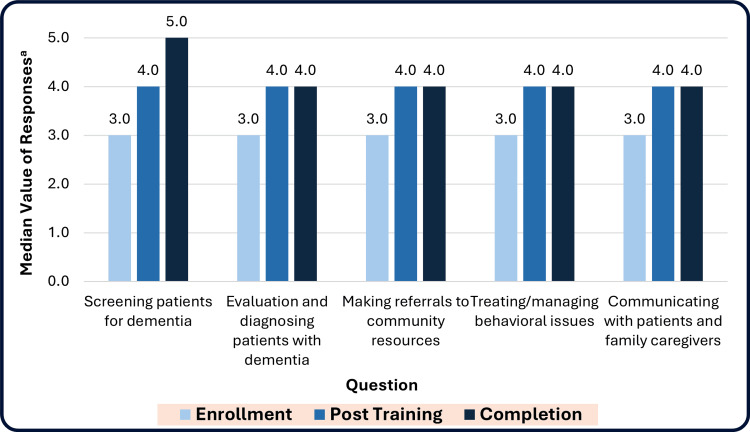
Median self-reported LPCP comfort with dementia-related clinical skills at enrollment, post-training, and completion among intervention group participants N=13 (100%) responding. Time periods: Post-training = approximately two months after enrollment; Completion = at the completion of the study (13-16 months after enrollment). LPCP: licensed primary care providers ^a^ Median comfort level calculated from response categories of (1=’Never’ or ‘Very Uncomfortable” to 5=’Always’ or ‘Extremely Comfortable’). The responses ranged from 1 to 5, with higher levels indicating greater experience or comfort (Enrollment response categories: 5 – extremely comfortable, 4 – moderately comfortable, 3 – somewhat comfortable, 2 – somewhat uncomfortable, 1 – very uncomfortable; and Completion response categories: 5 – always, 4 – usually, 3 – sometimes, 2 – rarely, 1 – never). Enrollment questions related to after any previous training, or at the time of enrollment if no previous training was completed.

LPCPs reported statistically significant increases in comfort providing dementia care and improved communication skills between enrollment and the post-training assessment on the five questions analyzed (Table 3, Figure 4). Most, between 12 (75.0%) and 15 (93.8%), reported improvements in the skills measured in each question between enrollment and the post-training assessment. These improvements were maintained to the study completion (13-16 months after enrollment) (Figure 4).

**Table 3:** Self-reported LPCP comfort with dementia-related clinical skills at enrollment (enroll) vs. post-training (post train), intervention group N=16 (100%). Post-training = time period after educational intervention (approximately two months after enrollment). Enrollment and post-training questions were compared using the Matched-Pairs Wilcoxon Signed-Rank test. The scale and subscales range from 1 to 5, with higher levels indicating greater experience or comfort (Enrollment response categories: 5 – extremely comfortable, 4 – moderately comfortable, 3 – somewhat comfortable, 2 – somewhat uncomfortable, 1 – very uncomfortable; Post-training response categories: 5 – always, 4 – usually, 3 – sometimes, 2 – rarely, 1 – never). Enrollment questions reported after any previous training, or at the time of the survey if no previous training was completed. LPCP: licensed primary care providers, Md: median, n: number of respondents with increase, p: p-value, Z: test statistic ^a^ Percentage of respondents with an increase in score between the enrollment and post-training time periods. *p<.01, **p<.001

Question (shortened)	Enroll	Post train	Z	p	Increase^a^	
	Md	Md	-	-	n	%
Comfortable screening for dementia	3.00	4.00	-3.40	< .001**	14	87.5
Comfortable evaluating and diagnosing dementia	3.00	4.00	-3.51	< .001**	15	93.8
Comfortable referring patients and family members to community resources	3.00	4.00	-3.42	< .001**	14	87.5
Comfortable managing behavioral issues (in persons with dementia)	3.00	4.00	-3.18	.001*	12	75.0
Communication skills with patients and family caregivers improved (post train)/comfort level (enroll)	3.00	4.00	-3.08	.002*	13	81.3

These same five questions increased at both clinical sites between enrollment and the post-training time period (Table 4).

**Table 4:** Self-reported LPCP comfort with dementia-related clinical skills at enrollment (enroll) vs. post-training (post train) per clinic site, intervention group N=16 (100%); 8 (100%) from each clinic. Enrollment and post-training questions were compared using the Matched-Pairs Wilcoxon Signed-Rank test per clinic site. Post-training = time period after educational intervention (approximately two months after enrollment). The scale and subscales range from 1 to 5, with higher levels indicating greater experience or comfort (Enrollment response categories: 5 – extremely comfortable, 4 – moderately comfortable, 3 – somewhat comfortable, 2 – somewhat uncomfortable, 1 – very uncomfortable; Post-training response categories: 5 – always, 4 – usually, 3 – sometimes, 2 – rarely, 1 – never). Enrollment questions reported after any previous training, or at the time of the survey if no previous training was completed. LPCP: licensed primary care providers, Md: median, n: number of respondents with increase ^a^ Percentage of respondents with an increase in score between the enrollment and post-training time periods. *p<.05

Question (shortened)	Scripps Ranch Clinic (Clinic 1)			Genesee Clinic (Clinic 2)		
	Enroll	Post-training	Increase^a^	Enroll	Post-training	Increase^a^
	Md	Md	n (%)	Md	Md	n (%)
Comfortable screening for dementia	3.00	4.50*	6 (75.0)	3.00	4.00*	8 (100.0)
Comfortable evaluating and diagnosing dementia	3.00	4.50*	8 (100)	3.00	4.00*	7 (87.5)
Comfortable referring patients and family members to community resources	3.00	4.00*	7 (87.5)	3.00	4.00*	7 (87.5)
Comfortable managing behavioral issues (in persons with dementia)	3.00	4.00*	6 (75.0)	3.00	3.50*	6 (75.0)
Communication skills with patients and family caregivers improved (post train)/comfort level (enroll)	3.00	4.00*	8 (100)	3.50	4.00	5 (62.5)

Self-reported clinical practices over time: enrollment, post-training, interval, and completion (LPCP intervention group)

LPCPs in the intervention group completed the CPS at three different periods: (1) Post-training - after the educational portion of the intervention (approximately two months after enrollment), (2) Interval - nine to 10 months after enrollment, and (3) Completion - after the study period concluded at each clinic (13 to 16 months after enrollment). Nineteen questions on the CPS were averaged to produce an overall mean (as these questions all measured knowledge, skills, and competence related to dementia care), the Clinical Practice Score. Five questions from the CIQ collected at enrollment were similar to the CPS questions and included in the analysis.

All of the LPCPs who responded (N=13, 100%) reported increased comfort in screening for dementia between enrollment and study completion, while 10 (76.9%) reported increased comfort evaluating and diagnosing dementia, referring patients and family to dementia resources, and managing behavioral issues with dementia patients (Table 5). Most, 10 (76.9%), also reported improved communication skills with dementia patients and their caregivers. The Clinical Practice Score increased for seven (53.8%) of the 13 LPCPs who completed the CPS at the post-training and completion time points, although this increase was not statistically significant (the Clinical Practice Score stayed the same for one (7.7%) of the LPCPs and decreased for five (38.5%)).

**Table 5:** LPCP self-reported responses on the clinical practice survey at enrollment (enroll), post-training, and completion time points, intervention group participants N=13 (100%) responding. Responses ranged from 1 to 5, with higher levels indicating a higher level of experience or comfort (Enrollment: 5 – extremely comfortable, 4 – moderately comfortable, 3 – somewhat comfortable, 2 – somewhat uncomfortable, 1 – very uncomfortable; Post-training, Interval and Completion: 5 – always, 4 – usually, 3 – sometimes, 2 – rarely, 1 – never). Enrollment questions reported after any previous training, or at the time of the survey if no previous training was completed. LPCP: licensed primary care providers, Md: median, n: number of respondents with increase, p: p-value, Z: test statistic Time periods: Post-training = approximately two months after enrollment; Completion = at the completion of the study (13-16 months after enrollment). ^a^ Median comparisons measured using the Matched-Pairs Wilcoxon Signed-Rank test for Completion vs. Post-training, or Completion vs. Enrollment if available. ^b^ Percentage of respondents with an increase in score between post-training and completion. ^c^ Mini-Cog; Eight-Item Informant Interview to Differentiate Aging and Dementia (AD8); Describe, Investigate, Create, and Evaluate (DICE). ^d^ Mean of responses to 19 questions on the LPCP Clinical Practice Survey (questions 2 through 20). *p<.05, **p<.01, ***P<.001

Question (shortened)	Enroll	Post-training	Completion	Z^a^	p^a^	Increase^b^	
	Md	Md	Md	-	-	n	%
Satisfied with the level of training on screening, diagnosis, and care management	-	5.00	4.00	-1.13	.257	1	7.7
Comfortable screening for dementia	3.00	4.00	5.00	-3.31	< .001***	13	100.0
Competent screening for dementia	-	4.00	5.00	-2.24	.025*	5	38.5
Comfortable evaluating and diagnosing dementia	3.00	4.00	4.00	-2.88	.004**	10	76.9
Competent evaluating and diagnosing dementia	-	4.00	4.00	0.00	>.999	2	15.4
Comfortable referring patients and family to resources	3.00	4.00	4.00	-2.66	.008**	10	76.9
Competent referring patients and family to resources	-	4.00	4.00	-.38	.705	3	23.0
Comfortable managing behavioral issues (persons with dementia)	3.00	4.00	4.00	-2.97	.003**	10	76.9
Competent managing behavioral issues (persons with dementia)	-	4.00	4.00	0.00	>.999	2	15.1
Use standardized screening and evaluation procedures in patients for whom dementia is a concern	-	5.00	4.00	-1.41	.157	0	0.0
Use the Mini-Cog^c^ as a screening instrument	-	4.00	4.00	-.79	.429	2	15.4
Use the AD8^c^ as a screening instrument	-	4.00	4.00	-.68	.496	3	23.1
Comfortable and confident using instrument chosen in my practice	-	5.00	5.00	-2.00	.046*	4	30.8
Investigate and treat reversible conditions before diagnosing cognitive impairment or referring to specialist	-	5.00	5.00	-1.00	.317	3	23.1
Incorporate DICE^c^ method into assessment and treatment for behavioral symptoms for dementia patients	-	4.00	3.00	-2.33	.020*	0	0.0
Educate caregivers on environmental and behavioral change interventions as one of my first steps	-	4.00	4.00	-1.89	.059	1	7.7
Utilize late-state dementia treatment practices	-	4.00	4.00	-.83	.405	6	46.2
Direct patients, caregivers, and clinicians to appropriate dementia care resources	-	4.00	4.00	0.00	>.999	3	23.1
Initiate advanced care planning conversations with patients and families early in the disease course	-	4.00	4.00	-1.41	.157	1	7.7
Communication skills with patients and family caregivers improved (comfort level asked at enrollment)	3.50	4.00	4.00	-2.97	.003**	10	76.9
Completion of Guidelines training made me more sensitive to the signs of cognitive decline in older patients	-	5.00	4.00	-1.34	.180	1	7.7
More aware of global reduction in my stress after mindfulness and self-compassion training	-	4.00	4.00	-1.27	.206	2	15.4
Experiences with patients have changed due to training in mindfulness and self-compassion	-	4.00	4.00	0.00	>.999	4	30.8
Clinical Practice Score^d^	-	4.16	4.21	-.59	.554	7	53.8

Statistically significant improvements were seen in six survey questions between enrollment (or post-training time period if no enrollment question) and study completion (Table 5). Five of the statistically significant increases were based on enrollment questions from the CIQ compared to study completion questions from the CPS. The response options differed between the two questionnaires (i.e., ‘extremely comfortable’ to ‘very uncomfortable’ versus ‘always’ to ‘never’) and may have affected the results.

Though not appearing in any of the tables included with the manuscript, data analysis revealed that changes in the Clinical Practice Score between the post-training time point and study completion did not vary by clinic site (Mann-Whitney U test for score difference, N= 13 (100%): median increase Scripps = .079 and median increase Genesee = .000, Z = -.14, p = .945). Similarly, the Clinical Practice Score at study completion did not vary between the clinic sites (Mann-Whitney U test for score difference, N = 13 (100%): median Scripps = 4.32 and median Genesee = 4.16, Z = -1.51, p = .138).

Three of the five CPS questions gathered at enrollment and study completion increased significantly at both clinic sites, and two questions were significant at one clinic but not the other (Table 6): (1) Comfort level referring patients and family caregivers to dementia resources at Scripps Ranch Clinic (Clinic 1); (2) Comfort level managing behavioral issues with dementia patients at Genesee Clinic (Clinic 2).

**Table 6:** LPCP self-reported responses to select questions on the clinical practice survey, enrollment (enroll) vs. completion time point by clinic site, intervention group Time periods: Enrollment = at enrollment into the intervention; Completion = completion of the study (13-16 months after enrollment). ^a^ Percentage of respondents with an increase in score between enrollment and completion time periods. *p<.05

Question (shortened)	Scripps Ranch Clinic			Genesee Clinic		
	Enroll	Completion	Increase^a^	Enroll	Completion	Increase^a^
	Md	Md	n (%)	Md	Md	n (%)
Comfortable screening for dementia	3.50	5.00*	6 (100.0)	3.00	5.00*	7 (100.0)
Comfortable evaluating and diagnosing dementia	3.50	5.00*	5 (83.3)	3.00	4.00*	5 (71.4)
Comfortable referring patients and family to community resources	3.00	4.00*	5 (83.3)	3.00	4.00	5 (71.4)
Comfortable managing behavioral issues with dementia patients	3.00	4.00	4 (66.7)	3.00	4.00*	6 (85.7)
Communication skills with patients and caregivers improved (comfort level asked on enrollment survey)	3.00	4.50*	5 (83.3)	4.00	4.00*	5 (71.4)

Self-reported clinical practice: LPCP intervention and LPCP comparison group

At the study completion, approximately 13-16 months after study enrollment at each clinic site, 10 (N=10, 100%) LPCPs who did not participate in the intervention (five, 50.0%, from each clinic site) completed the CPS as a comparison group. Their results were compared to those of 13 (N=13, 100%) LPCPs intervention group participants who completed the same survey at the same time point (seven, 53.9% from Clinic 2 and six, 46.2% from Clinic 1).

Intervention group LPCPs reported statistically significantly greater competence in referring dementia patients, family members, and clinicians to appropriate community/dementia care resources than comparison group LPCPs (Table 7). They were also statistically significantly more comfortable and competent in managing behavioral issues in persons with dementia, more likely to incorporate describe, investigate, create, and evaluate (DICE) methods into assessment and treatment of behavioral and psychological symptoms of dementia (BPSD), and more likely to report improved communication skills when compared to the comparison group LPCPs. Lastly, when examining all questions together (Modified Clinical Practice Score), the intervention group LPCPs reported statistically significantly greater levels of knowledge, skills, and competence than the comparison group LPCPs related to various aspects of dementia care.

**Table 7:** LPCP self-reported responses on the clinical practice survey at the completion time period, intervention group vs. comparison group N=20 to 23 (100%) responders. N=13 (100%) intervention group and N=10 (100%) comparison group, unless noted on specific questions (N=7-9, 100% responders). Median comparisons computed using the Mann-Whitney U test (intervention vs. comparison). Responses range from 1 to 5, with higher levels indicating a higher level of experience or comfort (5 – always, 4 – usually, 3 – sometimes, 2 – rarely, 1 – never). Time period: Completion = completion of the study (13-16 months after enrollment of intervention group participants). LPCP: licensed primary care providers, Md: median, N: number of respondents, p: p-value, Z: test statistic ^a^ Mean of 18 questions (questions 2-19) on the Clinical Practice Survey. ^b^ Mini-Cog; Eight-Item Informant Interview to Differentiate Aging and Dementia (AD8); Describe, Investigate, Create, and Evaluate (DICE). ^c^ Information not included due to few respondents (n=1 to 3). *p<.05, **p<.01

Question number	Question (shortened) and Clinical Practice Score^a^	LPCP group		N 100%	Z	p
		Intervention	Comparison			
		Md	Md			
1	Satisfied with level of training on screening, diagnosis, and care management (Comparison Group N=9 responders, 100%)	4.00	3.00	22	-1.72	.093
2	Comfortable screening for dementia	5.00	4.00	23	-1.61	.150
3	Competent screening for dementia	5.00	4.00	23	-1.48	.192
4	Comfortable evaluating and diagnosing dementia	4.00	4.00	23	-1.52	.174
5	Competent evaluating and diagnosing dementia	4.00	3.50	23	-.99	.236
6	Comfortable referring patients and family to resources	4.00	3.00	23	-1.49	.133
7	Competent referring patients and family to resources	4.00	3.00	23	-2.39	.012*
8	Comfortable managing behavioral issues (persons with dementia)	4.00	3.00	23	-2.40	.013*
9	Competent managing behavioral issues (persons with dementia)	4.00	3.00	23	-2.40	.013*
10	Use standardized screening and evaluation procedures in patients for whom dementia is a concern (Comparison Group N=9 responders, 100%)	4.00	4.00	22	-.08	>.999
11	Use the Mini-Cog^b^ as a screening instrument	4.00	2.50	23	-1.22	.257
12	Use the AD8^b^ as a screening instrument	4.00	4.00	23	-1.49	.136
13	Comfortable and confident using instrument chosen in my practice	5.00	4.50	23	-1.41	.166
14	Investigate and treat reversible conditions before diagnosing cognitive impairment or referring to specialist	5.00	5.00	23	-.45	.685
15	Incorporate DICE^b^ method into assessment and treatment for behavioral symptoms for dementia patients (Comparison Group N=9 responders, 100%)	3.00	1.00	22	-3.22	.001**
16	Educate caregivers on environmental and behavioral change interventions as one of my first steps	4.00	3.00	23	-1.27	.231
17	Utilize late-state dementia treatment practices	4.00	4.00	23	-.76	.471
18	Direct patients, caregivers, and clinicians to appropriate dementia care resources	4.00	3.00	23	-2.52	.017*
19	Initiate advanced care planning conversations with patients and families early in the disease course	4.00	4.00	23	-.32	.816
20	Communication skills with patients and family caregivers improved (Comparison Group – ‘as a result of the tools in EPIC EMR’) (Comparison Group N=7 responders, 100%)	4.00	2.00	20	-3.21	.001**
21	Completion of Guidelines training made me more sensitive to the signs of cognitive decline in older patients	4.00	-^c^	-	-	-
22	More aware of global reduction in my stress after mindfulness and self-compassion training	4.00	-^c^	-	-	-
23	Experiences with patients have changed due to training in mindfulness and self-compassion	4.00	-^c^	-	-	-
-	Modified Clinical Practice Score^a^	4.21	3.32	23	-2.55	.009**

The Modified Clinical Practice Score for each group (intervention and comparison) did not differ by clinic site: The Mann-Whitney U test for intervention group vs. clinic, N=13 (100%), median Scripps = 4.32 and median Genesee = 4.16, Z = -1.51, p = .146. The Mann-Whitney U test for comparison group vs. clinic, N=10 (100%), median Scripps = 3.68 and median Genesee = 3.26, Z = -.63, p = .595.

The Modified Clinical Practice Score comparison (intervention vs. comparison) per clinic site did not yield significant differences for either site, although this is likely due to the smaller per-clinic sample sizes: The Mann-Whitney U test for intervention vs. comparison at Scripps, N=11 (100%), median intervention score = 4.32 and median comparison score = 3.68, Z = -1.64, p = .126. The Mann-Whitney U test for intervention vs. comparison at Genesee, N=12 (100%), median intervention score = 4.16 and median comparison score = 3.26, Z = -1.95, p = .053.

In addition to the information in Table 7, comparison group participants reported having access to some of the new dementia-related care tools introduced at UCSD Health during this study. This access occurred because it was not technically possible to limit access to these tools to only those clinicians who were participating in LPCPs in this study.

Specifically, the comparison group participant (N=10, 100%) responses on the CPS revealed that 50.0% (n=5) participated in mindfulness training either through UCSD or another opportunity; 40.0% (n=4) utilized prompts and tools in EPIC to assess patients with memory concerns; 20.0% (n=2) accessed live or on-demand webinars available through the SDCAP CR or attended any live or virtual trainings on dementia provided by other sources; 10.0% (n=1) thought that their communications skills with patients and their family caregivers were ‘always’ improved as a result of online training or through the SDCAP CR (the other four responses were ‘sometimes,’ ‘rarely,’ and ‘never’).

Further analysis was undertaken to examine the differences in the percentage choosing ‘Always’ and ‘Usually’ responses on the CPS between the groups. While the CPS contains 23 questions in total, only 18 with similar phrasing were utilized for this analysis. The total number of questions where participants indicated that they ‘Always’ or ‘Usually’ feel or practice the skill was calculated.

Eighteen (18) of the 23 questions about competence, comfort, practice of skills, and use of screening instruments were examined for this analysis. At study completion, the LPCP intervention group responded ‘Always’ or ‘Usually’ on statistically significantly more of these questions than the LPCP comparison group (Table 8).

**Table 8:** Number of questions on the clinical practice survey with responses of ‘always’ or ‘usually’ at the completion time period, intervention vs. comparison group N=23 (100%) intervention and comparison groups. N=13 (100%) intervention group and N=10 (100%) comparison group. Median comparison computed using the Mann-Whitney U test (intervention vs. comparison). Md: median, N: number of respondents, p: p-value, Z: test statistic Time period: Completion = completion of the study (13-16 months after enrollment of intervention group participants). ^a^ Total of 18 questions examined (questions 2 through 19 on the Clinical Practice Survey). Question 20 was not included due to a lower response rate in the comparison group. Questions one, 21, 22, and 23 were not included as they differ in phrasing from the other questions. p<.01*

Number and median of questions with ‘always’ or ‘usually’ response^a ^	Intervention		Comparison		N	Z	P
	n	%	n	%	-	-	-
6 to 8 questions	1	7.7	4	40.0	-	-	-
9 to 10 questions	1	7.7	3	30.0	-	-	-
11 to 12 questions	1	7.7	1	10.0	-	-	-
13 to 14 questions	0	0.0	1	10.0	-	-	-
15 to 16 questions	5	38.5	0	0.0	-	-	-
17 to 18 questions	5	38.5	1	10.0	-	-	-
Median number of questions with ‘always’/’usually’ responses^a^	Md 16.00		Md 9.00		23 (100%)	-2.69	.006*

**Figure 5:**
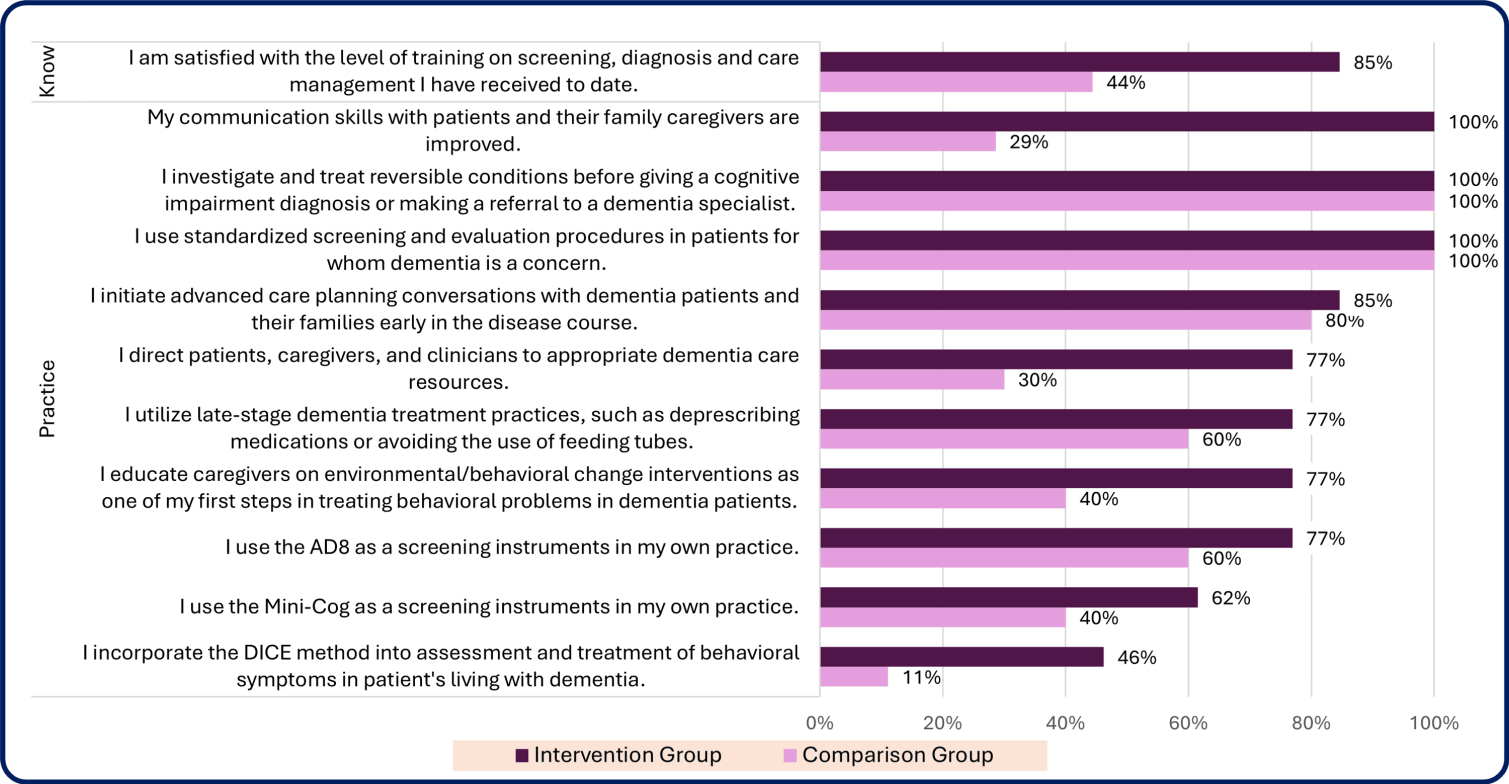
Percentage responding ‘always/usually’ to knowledge and practice questions from the clinical practice survey at completion, intervention group and comparison group N=13 (100%) intervention group, and N=7-10 (100%) comparison group. The questions and median range from 1 to 5, with higher levels indicating a higher level of experience or comfort (Response categories: 5 – always, 4 – usually, 3 – sometimes, 2 – rarely, 1 – never). n = number of respondents reporting ‘usually’ or ‘always,’ % = percentage of respondents. Time period: Completion = completion of the study (13-16 months after enrollment of intervention group participants).

**Figure 6:**
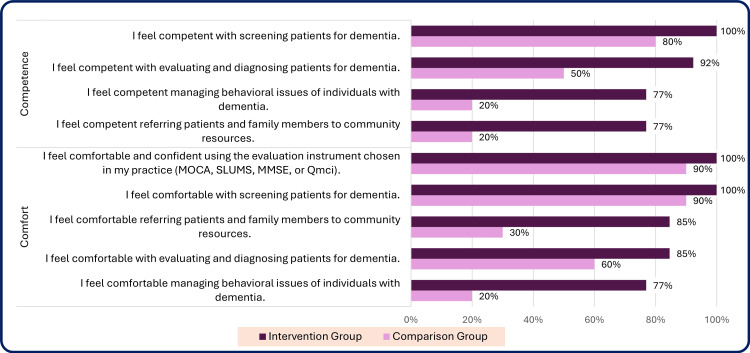
Percentage responding ‘always/usually’ to comfort and competence questions from the clinical practice survey at completion, intervention group vs. comparison group N=13 (100%) intervention group and N=10 (100%) comparison group participants. The questions and median range from 1 to 5, with higher levels indicating a higher level of experience or comfort (Response categories: 5 – always, 4 – usually, 3 – sometimes, 2 – rarely, 1 – never). n = number of respondents reporting ‘usually’ or ‘always,’ % = percentage of respondents. Time period: Completion = completion of the study (13-16 months after enrollment of intervention group participants).

A comparison of the number and percentage of participants who responded ‘Always’ or ‘Usually’ for each question on the CPS by group is shown in Table 9 and Figures 5, 6.

**Table 9:** Percentage responding ‘always/usually’ to questions from the clinical practice survey at the completion time period among intervention group and comparison group respondents N=13 (100%) intervention group and N=10 (100%) comparison group, unless noted on specific questions (N=7-9, 100% responders). The questions and median range from 1 to 5, with higher levels indicating a higher level of experience or comfort (Response categories: 5 – always, 4 – usually, 3 – sometimes, 2 – rarely, 1 – never). Time period: Completion = completion of the study (13-16 months after enrollment of intervention group participants). n: number of respondents reporting ‘usually’ or ‘always,’ %: percentage of respondents reporting ‘usually’ or ‘always’. ^a^ Mini-Cog; Eight-Item Informant Interview to Differentiate Aging and Dementia (AD8); Describe, Investigate, Create, and Evaluate (DICE). ^b^ Information not included due to few respondents (n=1 to 3).

Question number	Shortened question (sample size if other than indicated)	Intervention		Comparison	
		n	%	n	%
1	Satisfied with level of training on screening, diagnosis, and care management (Comparison Group N=9 responders, 100%)	11	84.6	4	44.4
2	Comfortable screening for dementia	13	100.0	9	90.0
3	Competent screening for dementia	13	100.0	8	80.0
4	Comfortable evaluating and diagnosing dementia	11	84.6	6	60.0
5	Competent evaluating and diagnosing dementia	12	92.3	5	50.0
6	Comfortable referring patients and family to resources	11	84.6	3	30.0
7	Competent referring patients and family to resources	10	76.9	2	20.0
8	Comfortable managing behavioral issues (persons with dementia)	10	76.9	2	20.0
9	Competent managing behavioral issues (persons with dementia)	10	76.9	2	20.0
10	Use standardized screening and evaluation procedures in patients for whom dementia is a concern (Comparison Group N=9 responders, 100%)	13	100.0	9	100.0
11	Use the Mini-Cog^a^ as a screening instrument	8	61.5	4	40.0
12	Use the AD8^a^ as a screening instrument	10	76.9	6	60.0
13	Comfortable and confident using instrument chosen in my practice	13	100.0	9	90.0
14	Investigate and treat reversible conditions before diagnosing cognitive impairment or referring to specialist	13	100.0	10	100.0
15	Incorporate DICE^a^ method into assessment and treatment for behavioral symptoms for dementia patients (Comparison Group N=9 responders, 100%)	6	46.2	1	11.1
16	Educate caregivers on environmental and behavioral change interventions as one of my first steps	10	76.9	4	40.0
17	Utilize late-state dementia treatment practices	10	76.9	6	60.0
18	Direct patients, caregivers, and clinicians to appropriate dementia care resources	10	76.9	3	30.0
19	Initiate advanced care planning conversations with patients and families early in the disease course	11	84.6	8	80.0
20	Communication skills with patients and family caregivers improved [Comparison – ‘as a result of the tools in EPIC EMR’] (Comparison Group N=7 responders, 100%)	13	100.0	2	28.6
21	Completion of Guidelines training made me more sensitive to the signs of cognitive decline in older patients	12	92.3	-^b^	-
22	More aware of global reduction in my stress after mindfulness and self-compassion training	7	53.8	-^b^	-
23	Experiences with patients have changed due to training in mindfulness and self-compassion	9	69.2	-^b^	-

Assessment of clinician adherence to the SDCAP screening algorithm

Integration of the SDCAP Screening Algorithm into the EPIC EMR provided objective data regarding the behavior of both participating and non-participating LPCPs related to the dementia screening algorithm. Data in EPIC flowsheets recorded how often patients completed AD8 screening for those with screening due, confirmed that a patient was “up to date” with AD8 screening (i.e., completed within the past 12 months), and if a MoCA or SLUMS was completed within the first two visits following a positive AD8 screening.

**Figure 7:**
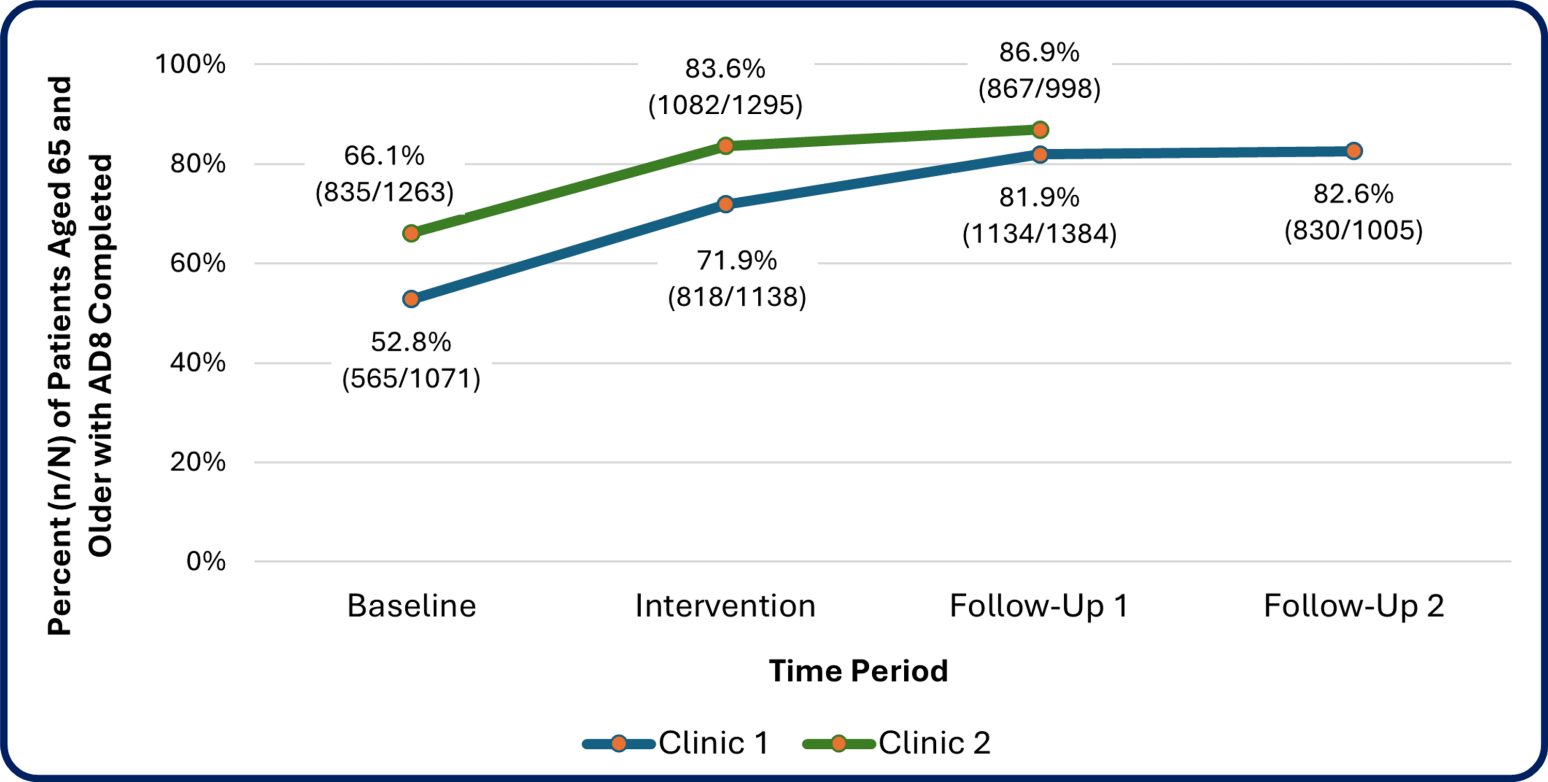
Percentage of patients aged 65 and older with AD8 screening completed in the past 12 months (“up-to-date”) by clinic site across time periods Numbers with up-to-date screening/N aged 65 and older included for each percentage. Time periods: Baseline (Clinic 1 (Scripps Ranch) 11-1-22 – 4/30/23 and Clinic 2 (Genesee) 5/1/23 to 10/31/23); Intervention (Clinic 1 (Scripps Ranch) 5/1/23 – 10/31/23 and Clinic 2 (Genesee) 1/1/24 – 6/30/24); Follow-Up 1 (Clinic 1 (Scripps Ranch) 11/1/23 – 6/30/24 and Clinic 2 (Genesee) 7/1/24 – 10/15/24); Follow-Up 2 (Clinic 1 7/1/24 – 10/15/24 and not yet available for Clinic 2 (Genesee)).

Information about patients aged 65 and older from EPIC was examined pre-study, baseline at start of the study, during intervention, and two post-intervention follow-up time points. An analysis of these data revealed that AD8 screening rates increased significantly between the pre-study and follow-up time periods (Tables 10, 11, and Figure 7). Clinic 1 screening rates increased from 31.2% (n=220) of those due for a yearly AD8 screening at baseline to 78.5% (n=299) at the latest post-intervention period, X2 (1, N=1086) = 221.51, p<.001. Clinic 2 screening rates increased from 56.0% (n=448) of those due for a yearly AD8 screening at baseline to 94.1% (n=349) at the post-intervention period, X2 (1, N=1171) = 168.99, p<.001. The percentage of patients aged 65+ with an AD8 screening completed in the past 12 months (i.e., “up-to-date”) at Scripps increased from 52.8% (n=565) at baseline to 82.6% (n=830) at the latest post-intervention period, X2 (1, N=2076) = 209.34, p<.001. The percentage of patients aged 65+ with an AD8 screening completed in the past 12 months (i.e., “up-to-date”) at Genesee increased from 66.1% (n=835) at baseline to 86.9% (n=867) at the post-intervention period, X2 (1, N=2261) = 129.11, p<.001.

**Table 10:** Percentage of patients aged 65 and older with AD8 screening completed out of those due for screening by clinic site across time periods, baseline vs. latest follow-up Comparisons calculated using Pearson Chi-square test; degrees of freedom = 1. Time periods: Baseline (Clinic 1 11-1-22 – 4/30/23 and Clinic 2 5/1/23 to 10/31/23); Intervention (Clinic 1 5/1/23 – 10/31/23 and Clinic 2 1/1/24 – 6/30/24); Follow-up 1 (Clinic 1 11/1/23 – 6/30/24 and Clinic 2 7/1/24 – 10/15/24); Follow-up 2 (Clinic 1 7/1/24 – 10/15/24 and not yet available for Clinic 2). N: number of patients aged 65+ due for AD8 screening, screened, n: number of persons screened, %: percentage screened, N2: sample size for statistical test (baseline vs. follow-up 2 for Clinic 1 and baseline vs. follow-up 1 for Clinic 2), p: p-value, X2: test statistic *p<.001

Time period	Scripps Ranch Clinic (Clinic 1)						Genesee Clinic (Clinic 2)					
	Patients due	AD8 completed		N^2^	X^2^	p	Patients due	AD8 completed		N^2^	X^2^	p
	N	n	%				N	n	%			
Baseline	705	220	31.2	1086 (100%)	221.51	< .001*	800	448	56.0	1171 (100%)	168.99	< .001*
Intervention	725	486	67.0	-	-	-	738	643	87.1	-	-	-
Follow-up 1	777	642	82.6	-	-	-	371	349	94.1	-	-	-
Follow-up 2	381	299	78.5	-	-	-	-	-	-	-	-	-

**Table 11:** Percentage of patients aged 65 and older with AD8 screening completed in the past 12 months (“up-to-date”) by clinic site across time periods, baseline vs. latest follow-up Comparisons calculated using Pearson Chi-square test; degrees of freedom = 1. Time periods: Baseline (Clinic 1 11-1-22 – 4/30/23 and Clinic 2 5/1/23 to 10/31/23); Intervention (Clinic 1 5/1/23 – 10/31/23 and Clinic 2 1/1/24 – 6/30/24); Follow-Up 1 (Clinic 1 11/1/23 – 6/30/24 and Clinic 2 7/1/24 – 10/15/24); Follow-Up 2 (Clinic 1 7/1/24 – 10/15/24 and not yet available for Clinic 2). N: number of patients aged 65+, screened, n: number of persons screened, %: percentage screened, N2: sample size for statistical test (baseline vs. follow-up 2 for Clinic 1 and baseline vs. follow-up 1 for Clinic 2), p: p-value, X2: test statistic *p<.001

Time period	Scripps Ranch Clinic (Clinic 1)						Genesee Clinic (Clinic 2)					
	Patients due	AD8 completed		N^2^	X^2^	p	Patients due	AD8 completed		N^2^	X^2^	p
	N	n	%				N	n	%			
Baseline	1071	565	52.8	2076 (100%)	209.34	< .001***	1263	835	66.1	2261 (100%)	129.11	< .001*
Intervention	1138	818	71.9	-	-	-	1295	1082	83.6	-	-	-
Follow-up 1	1384	1134	81.9	-	-	-	998	867	86.9	-	-	-
Follow-up 2	1005	830	82.6	-	-	-	-	-	-	-	-	-

## Discussion

Primary care providers see their patients over the course of many years for a range of health issues and are considered to have the best vantage point for observing changes in their patients. It is therefore critical that LPCPs have the necessary tools to effectively screen, diagnose, and provide care for individuals impacted by dementia and the family members who care for them. Research reveals that a substantial portion of those who would meet the diagnostic criteria for Alzheimer’s and other dementias have not been diagnosed with dementia by a physician [1].

Centers for Medicare and Medicaid Services, which created the Medicare Annual Wellness Visit (AWV), mandates that the visit should include an assessment of an individual’s cognitive function, but does not specify screening instruments or a workflow [3]. Research has revealed that the AWV is underused, with only 23 to 34% of those eligible experiencing it [4,5]. When AWVs do take place, fewer than 30% include a cognitive assessment [6]. Other studies have highlighted the importance of provider education for improving screening rates [20]. Prior to this study, large healthcare systems in San Diego lacked tools in their EMR to integrate cognitive evaluation in a streamlined fashion. In analyzing the issue, this study has identified avenues to improve screening rates while also helping PCPs grow more confident in their response to screening results. 

Although 13 (81.3%) of intervention group PCPs had received some level of training on dementia during the four years prior to entering this study, they reported statistically significant increases in comfort and competence in providing dementia care after the SDCAP training. When the intervention group was compared to the comparison group at study completion, the intervention group reported statistically significantly greater levels of skill, comfort, and competence in various components of dementia care. These observations demonstrate both the need for PCPs to have additional dementia training, in general, as well as the effectiveness of the training provided through this study, in particular.

On the CIQ instrument, 4 (40.0%) of comparison group PCPs reported utilizing prompts and tools in EPIC to assess patients with memory concerns, and two (20.0%) reported previous (within the past four years) dementia training through online webinars and/or live or virtual dementia care training, including those provided by SDCAP. Their access to EPIC tools occurred because it was not technically possible to limit access to these tools to only clinicians who were participating in the study. Comparison group PCPs' participation in SDCAP educational experiences occurred because educational training through the SDCAP has been ongoing since 2017. If the comparison group PCPs had not had access to the EPIC tools and/or none had participated in SDCAP training and educational programs, the differences reported between the intervention group and the comparison group may have been even greater.

A couple of factors make the increase in the AD8 screening rate in this study especially noteworthy. The AD8 screening rate during the pre-study period was not zero. As previously noted, EPIC at UC San Diego Health had been modified prior to the launch of this study: the AWV automatically included an AD8, and some screening had been occurring prior to the launch of this study, albeit without any of the additional automated decision support tools in EPIC that this study created. In addition, the calculations of screening rates during the pre- study period included patients being cared for by both intervention group and comparison group PCPs.

This study has a number of weaknesses, including the small sample size, the exclusive reliance on subjective measures of comfort and competence, the possibility of measurement bias when using subjective outcomes, the additional modifications of EPIC made after the study launched, and comparison group contamination, as they also had access to the EPIC modifications. Additionally, a selection bias could be viewed as a weakness of the study, given that participation in the study at the clinics was voluntary on the part of the participating LPCPs. Additionally, a weakness is inherent in the use of the participant-completed AD8; due to factors such as anosognosia, participant-completed AD8s are less reliable than if a caregiver completes the survey and can, therefore, result in false negatives [21].

It is of note that the comparison group disclosed accessing the new care tools (i.e., Grand Rounds and online training, EPIC tools, mindfulness training, etc.). Limiting access to these tools was judged not to be ethical because the tools were created with the intention of improving the quality of care and could potentially benefit any patient receiving care at UCSD Health; however, if this had not occurred, the differences in knowledge and skills reported between the intervention and comparison groups would likely have been greater than reported. Educational training by SDCAP has been ongoing since 2017, and 8 (80.0%) of the comparison group participants reported at least one of the following on the study completion CPS: (1) utilizing tools and prompts in EPIC to assess patients with memory concerns, (2) participating in mindfulness training either through UCSD or another opportunity, and/or (3) accessing online webinars available through the SDCAP CR or attending any live or virtual trainings on dementia. It is, therefore, also likely that the additional intervention components and greater intervention dosage received by the intervention group led to the measurable improvements in dementia care knowledge, skills, and competence that surpassed the gains reported by the comparison group LPCPs.

A major strength of this study was the goal of enhancing dementia care in primary care by evaluating the usefulness of targeted training in dementia care, screening algorithms, and enhanced EMR-based workflows, which takes into account the very limited time available to LPCPs to undertake dementia screening, while also meeting clinical productivity expectations. Other strengths of this study included the use of online learning modules, which LPCPs could complete according to their respective schedules, the use of online data collection instruments, and the ability to make significant changes in EPIC to support the goals of this study. The study led to making a case for the importance of EMR integration of screening tools, and since completion, the SDCAP CR has helped five other health systems/divisions in San Diego adopt standards of practice and EMR tools for cognitive screening and evaluation [12].

## Conclusions

Other healthcare experts have suggested population health solutions for detecting cognitive impairment in older adults. In 2022, after the launch of this study, the National Academy of Neuropsychology (NAN) hosted an interdisciplinary meeting of representatives of national organizations committed to improving care of older adults. This group created and published a workflow for detection of cognitive impairment during routine primary care visits similar to the one developed by SDCAP. Both start with the use of a risk stratification tool administered as part of the Medicare AWV. Similarly, the State of California launched the Dementia Care Aware initiative utilizing an almost-identical algorithm to increase screening (and associated billing) for Medicaid-only eligible older adults. Ongoing research will be essential in validating the results of this study and will help answer important remaining questions such as whether increased utilization of mid-level providers, computerized cognitive screening tools, or other technological advances will enhance dementia screening and care provided by LPCPs.

## Appendices

Appendix A

Clinician Initial Questionnaire (CIQ)

1) Have you received any training on dementia in the past four years?

a) Yes b) No

2) If yes, please indicate the most recent training:

a) Within the past month b) 2 - 6 months ago c) 7 - 12 months ago d) More than one year ago

3) If yes, please indicate the subject of the training you received - (check all)

- Program overview - Screening patients with cognitive declines - Evaluation and diagnosis - Behavioral management - Pharmacotherapy - Pharmacological management of behavioral issues - End-of-life issues - Patient and caregiver communications - Mindfulness/self-care training

4) Method of training you received

- Grand Rounds/presentations live webinar or in-person at your health system - Professional conference/meeting - Online recorded webinar - Read Clinical Guidelines booklet - Reviewed championsforhealth.org/alzheimers website - Other

5) In your practice, do you perform the Medicare Annual Wellness Visit?

a) Yes b) No

6) In your practice, do you conduct screenings on patients for suspected dementia?

a) Yes b) No

7) In your practice, how often do you screen for dementia among patients 65 or older?

a) When the patient or family member requests b) As part of an annual wellness exam c) About 10% of patients d) About 25% of patients e) About 50% of patients f) About 75% of patients

8) In your current practice, how often do your patients who you screen require a complete evaluation for dementia?

a) About 10% of patients screened b) About 25% of patients c) About 50% of patients d) About 75% of patients e) Unsure

9) Do you conduct the evaluation or refer to a specialist?

a) Conduct evaluation b) Refer to specialist

10) In an average month, what percent of your patient visits are with patients 65+?

a) Less than 10% b) 11-25% c) 26-50% d) 50-75% e) More than 75%

11) Before the training you received in the past four years, or now, if you did not receive specific training, how comfortable were you with: (Please answer each using the following guide: Always = 5, Usually = 4, Sometimes = 3, Rarely = 2, Never = 1)

a) Screening for dementia b) Diagnosing dementia c) Referring to community resources d) Treating Behavioral Symptoms e) Communicating with patients and family caregivers on dementia

12) After the training you received, how comfortable were you with: (Please answer each using the following guide: Always = 5, Usually = 4, Sometimes = 3, Rarely = 2, Never = 1)

a) Screening for dementia b) Diagnosing dementia c) Referring to community resources d) Treating Behavioral Symptoms e) Communicating with patients and family caregivers on dementia

13) Up till now, have you incorporated any of the Guidelines in your practice?

a) Yes b) No

14) If yes, please indicate which ones:

a) Screening algorithm b) Use of Mini-Cog and/or AD8 c) Evaluation algorithm d) Use of MoCA, SLUMS or MMSE instrument e) Informant surveys f) Depression screening g) Behavioral management h) Pharmacological management of behavioral signs and symptoms i) Life planning through the stages of disease, including end-of-life planning

15) How many times have you used any of the Guidelines?

a) Never b) 1-5 times c) 6-15 times d) More than 15 times e) With the majority of my patients presenting with cognitive issues f) For most/all patients 65+

16) Professional degree: MD, DO, RN, NP, Medical Resident

17) Type of practice: primary care, family medicine, internal medicine, neurology, psychiatry, emergency department

18) How long have you been a primary care provider?

a) Less than five years b) Five to 10 years c) More than 10 years

19) How would you describe yourself?

a) American Indian or Alaska Native b) Asian c) Black or African American d) Middle Eastern or North African e) Native Hawaiian or other Pacific Islander f) White g) Hispanic or Latino h) Not Hispanic or Latino i) Prefer not to state

20) Gender

a) Male b) Female c) Non-binary d) Choose not to disclose

Appendix B

Clinical Practice Survey (CPS) - Post-Training Interval Survey

Please answer these questions using the following guide:

Always = 5, Usually = 4, Sometimes = 3, Rarely = 2, Never= 1

1) I am satisfied with the level of training on screening, diagnosis, and care management I have received to date.

2) I feel comfortable and competent with screening patients for dementia.

3) I feel comfortable and competent with evaluating and diagnosing patients for dementia.

4) I feel comfortable and competent referring patients and family members to community resources.

5) I feel comfortable and competent managing behavioral issues of individuals with dementia.

6) I use standardized screening and evaluation procedures in patients for whom dementia is a concern.

7) I use the Mini-Cog, AD8, or PHQ-2 screening instruments in my own practice.

8) I feel comfortable and confident using the evaluation instrument chosen in my practice (MoCA, SLUMS, MMSE, or Qmci).

9) I investigate and treat reversible conditions before giving a cognitive impairment diagnosis or making a referral to a dementia specialist.

10) I incorporate the DICE method into the assessment and treatment of behavioral symptoms in patients living with dementia.

11) I educate caregivers on environmental and behavioral change interventions as one of my first steps in treating behavioral problems in patients living with dementia.

12) I utilize late-stage dementia treatment practices, such as deprescribing medications or avoiding the use of feeding tubes.

13) I direct patients, caregivers, and clinicians to appropriate dementia care resources.

14) I initiate advanced care planning conversations with dementia patients and their families early in the disease course.

15) My communication skills with patients and their family caregivers have improved
